# Draft Genome Sequence of *Desulfofundulus thermobenzoicus* subsp. *thermosyntrophicus* DSM 14055, a Moderately Thermophilic Sulfate Reducer

**DOI:** 10.1128/MRA.01416-19

**Published:** 2020-01-16

**Authors:** Emma Bertran, Lewis M. Ward, David T. Johnston

**Affiliations:** a Department of Earth and Planetary Sciences, Harvard University, Cambridge, Massachusetts, USA; b Department of Geosciences, Princeton University, Princeton, New Jersey, USA; c Earth-Life Science Institute, Tokyo Institute of Technology, Tokyo, Japan; University of Southern California

## Abstract

Here, we describe the genome of Desulfofundulus thermobenzoicus subsp. *thermosyntrophicus* DSM 14055, a member of the Clostridiales that is capable of sulfate reduction coupled to the oxidation of propionate, lactate, pyruvate, and H_2_/CO_2_. This genome expands our understanding of microbial sulfate reduction (MSR) in anaerobic methanogenic environments.

## ANNOUNCEMENT

*Desulfofundulus thermobenzoicus* subsp. *thermosyntrophicus* DSM 14055 is a thermophilic bacterium first isolated from methanogenic sludge in coculture with Methanobacterium thermoautotrophicum Z245 (1). It belongs to the *Clostridiales* order within the bacterial domain and grows most optimally anaerobically at 55°C and a pH of 7 to 7.51. When grown syntrophically with methanogens, D. thermobenzoicus grows as a propionate oxidizer. In pure culture, *D. thermobenzoicus* grows by fermentation of benzoate, fumarate, H_2_/CO_2_, pyruvate, and lactate, coupled to propionate oxidation to sulfate reduction (1). Microbial sulfate reduction (MSR) has been suggested as a regulatory mechanism for methanogenesis in modern environments. This genome sequence allows refinement of our grasp of the biochemistry of sulfate-reducing organisms that grow syntrophically with methanogens.

Purified genomic DNA for *D. thermobenzoicus* subsp. *thermosyntrophicus* DSM 14055 was received from the DSMZ. Cultures were grown in medium 684, and DNA was extracted with a MasterPure Gram-positive DNA purification kit from Epicentre. Genomic DNA libraries were prepared using a Nextera XT library prep kit on a Hamilton Microlab Star automated liquid-handling system. Sequencing was performed via the Illumina HiSeq platform using a 250-bp paired-end protocol. Adapter trimming was performed with Trimmomatic v0.30 (2), and *de novo* assembly and annotation were performed using SPAdes v3.7 (3) and RAST v2.0 (4), respectively. The publicly available genome was annotated with PGAP (5). We used CheckM v1.0.12 (6) to estimate genome completeness, and MetaPOAP v1.0 (7) to estimate the likelihood of the presence or absence of metabolic pathways. Taxonomic assignment of the genome was verified with GTDB-Tk v0.3.2 (8). Default parameters were used for all software unless otherwise specified.

Sequencing coverage was ∼170× and resulted in 1,596,007 reads assembled into a draft genome consisting of 303 contigs with an *N*_50_ value of 58,756 bp and totaling 3,717,957 bp, which encoded 4,357 coding sequences and 55 RNAs. The genome has a 55.8% GC content. CheckM estimates the genome to be 100% complete based on the presence of conserved single-copy marker genes with 2.58% redundancy and 0% strain heterogeneity.

Dissimilatory sulfate reducers typically encode a conserved set of enzymes that include sulfate adenylyltransferase (DsrABC), adenylylsulfate reductase (AprAB), and the sulfite reduction-associated DsrMKJOP complex (9). The genes encoding these enzymes were largely recovered in the *D. thermobenzoicus* genome, with the notable exceptions of *dsrM*, *dsrJ*, *dsrO*, and *dsrP.* The incomplete nature of this genome and the typically scattered organization (i.e., the lack of conserved operon structure) of the genes encoding the DsrMKJOP complex make it difficult to determine whether these genes were simply not recovered in the draft genome presented here or if they are actually absent from the *D. thermobenzoicus* genome.

The taxonomic and ecological distribution and genetic diversity of dissimilatory sulfate-reducing microorganisms remain topics of significant interest, with major relevance for understanding biogeochemical cycles (10, 11), including the regulation of methanogenesis in sedimentary environments (11–13). The genome of *D. thermobenzoicus* is therefore valuable for expanding our understanding of sulfate reducers capable of syntrophic growth with methanogens, as well as for improving the genomic representation of sulfate-reducing *Firmicutes*.

### Data availability.

This whole-genome shotgun project has been deposited at DDBJ/ENA/GenBank under the accession number WHYR00000000. The FASTQ files of the raw reads were deposited in the NCBI SRA under accession number SRR10430293.
